# Exploring Adherence to Pelvic Floor Muscle Training in Women Using Mobile Apps: Scoping Review

**DOI:** 10.2196/45947

**Published:** 2023-11-30

**Authors:** Rosie C Harper, Sally Sheppard, Carly Stewart, Carol J Clark

**Affiliations:** 1 Faculty of Health and Social Sciences Bournemouth University Bournemouth United Kingdom; 2 National Institute of Health Research ARC Wessex Southampton United Kingdom; 3 University Hospitals Dorset NHS Foundation Trust Poole United Kingdom; 4 Faculty of Science and Technology Bournemouth University Bournemouth United Kingdom

**Keywords:** adherence, behavior change, mHealth, mobile apps, pelvic floor muscle training, women

## Abstract

**Background:**

Pelvic floor dysfunction is a public health issue, with 1 in 3 women experiencing symptoms at some point in their lifetime. The gold standard of treatment for pelvic floor dysfunction is supervised pelvic floor muscle training (PFMT); however, adherence to PFMT in women is poor. Mobile apps are increasingly being used in the National Health Service to enable equity in the distribution of health care and increase accessibility to services. However, it is unclear how PFMT mobile apps influence PFMT adherence in women.

**Objective:**

We aimed to identify which behavior change techniques (BCTs) have been used in PFMT mobile apps, to distinguish the core “capability, opportunity, and motivation” (COM) behaviors targeted by the BCTs used in PFMT mobile apps, and to compare the levels of PFMT adherence in women between those using PFMT mobile apps and those receiving usual care.

**Methods:**

We conducted a scoping review of the literature. Published quantitative literature that compared the use of a PFMT mobile app to a control group was included to address the objectives of the study. The electronic bibliographic databases searched included MEDLINE, CINAHL, Scopus, Web of Science, and PEDro, along with CENTRAL. Studies were also identified from reference searching of systematic reviews. Original articles written in English from 2006 onward were included. Nonexperimental quantitative studies, qualitative studies, studies that use male participants, case studies, web-based interventions, and interventions that use vaginal probes were excluded. Narrative synthesis was conducted on eligible articles based on the aims of the study.

**Results:**

Of the 114 records retrieved from the search, a total of 6 articles met the eligibility and inclusion criteria. The total number of participants in the studies was 471. All PFMT mobile apps used the BCT “prompts and cues.” Opportunity was the core COM behavior targeted by the PFMT mobile apps. Higher levels of adherence to PFMT were observed among women using PFMT mobile apps.

**Conclusions:**

Digital “prompts and cues” are a BCT commonly used in PFMT mobile apps, and further research is required to practically assess whether a future randomized controlled trial that investigates the effectiveness of digital “prompts and cues” on PFMT adherence in women can be conducted.

## Introduction

Pelvic floor dysfunction (PFD) is a public health issue, with 1 in 3 women experiencing symptoms at some point in their lifetime [1,2]. PFD is a long-term condition that can worsen or reoccur in women at predictable points in their lives, including the childbearing years, menopause, and older age [2].

Symptoms of PFD include urinary incontinence (including mixed, urge, and stress urinary incontinence), pelvic organ prolapse, fecal incontinence, and sexual dysfunction, with stress urinary incontinence (SUI) as the most prevalent symptom [3,4]. SUI is defined by the International Continence Society as “the complaint of any involuntary loss of urine on effort or physical exertion or on sneezing or coughing” [5]. The gold standard of prevention and treatment for SUI is supervised pelvic floor muscle training (PFMT) [2,6,7]. Adherence to PFMT is key to its effectiveness in the treatment of symptoms of PFD [7,8]. The evidence suggests PFMT for a minimum of 3 months reduces symptoms of PFD and increases the likelihood of long-term adherence [2,9].

Adherence in relation to exercise prescription is the commitment with which a person sticks to a prescribed regime, which may be registered in terms of their compliance with the regime [7,10]. Exercise adherence can be measured in multiple ways, including exercise diaries [11,12] and exercise session attendance [13]. Self-efficacy, an individual’s belief in their own capability to successfully execute specific actions to attain certain outcomes, has been suggested to be a key determinant in women’s short- and long-term adherence to PFMT [12]. Adherence to PFMT in women is key to preventing and treating symptoms of PFD [12]. However, women’s adherence to PFMT is poor and is associated with forgetting to complete PFMT [14].

Self-efficacy is suggested to be a key factor influencing women’s short- and long-term adherence to PFMT [12]. Pelvic health physiotherapists use behavior change techniques (BCT) to increase women’s self-efficacy to facilitate engagement with PFMT [2]. However, the effectiveness of these techniques is hindered by limited access to services and delayed continuity of care [15]. Furthermore, the impact of COVID-19 on health care services has exacerbated the issue, as the redirection of resources through the suspension of outpatient services has led to a backlog of patients awaiting specialist care [16].

Digital health technology is being progressively integrated into the National Health Service to enable equitable distribution of health care amidst ever-growing pressures [17]. Among these technologies, mobile apps have gained prominence as valuable tools for self-management, particularly among individuals with long-term conditions [18]. Mobile apps offer convenient and timely health information delivery, comparing favorably with web-based interventions [18-21]. In the current health care landscape, marked by challenges to service delivery, mobile apps offer a promising pathway for facilitating PFMT interventions. Apps incorporating effective BCTs should actively guide and support women in their PFMT and maximize adherence.

By identifying the BCTs used within PFMT mobile apps and the core behaviors they address, it is expected that future digital interventions aimed at improving PFMT adherence in women could be designed to focus on the most effective core behavior. The Behavior Change Wheel (BCW) is a theoretically underpinned framework using 19 different behavioral science frameworks and provides a standardized taxonomy for the characteristics of behavioral interventions [22]. The Capability, Opportunity, Motivation, Behavior (COM-B) model sits at the core of the BCW and names the 3 key elements of behavior change: capability, opportunity, and motivation. The Capability, Opportunity, Motivation, Behavior Change Wheel (COM-BCW) links the theoretical domains framework with the theoretical models of behavior. This means behavioral interventions can be characterized and evaluated using the COM-BCW through the identification of BCTs [23,24]. Experts in PFMT adherence have recommended using the COM-B to map the behavioral theories underpinning interventions to improve PFMT adherence in women [7].

Some systematic review findings suggest that PFMT mobile apps increase PFMT adherence in women [8,21,25,26] and their effectiveness in the treatment of PFD [27]. However, it is unclear how they work. The aim of one study [28] was to determine the encouraging features of PFMT mobile apps using persuasive system design to suggest features for new PFMT mobile apps that incorporate the COM-B model. However, to date, no review has mapped the core behaviors targeted by PFMT mobile apps through the identification of BCTs using the COM-B model. The findings of this scoping review will add to the body of literature around the theoretical underpinnings of existing interventions used in PFMT mobile apps and their influence on women’s adherence to PFMT compared with usual care.

Therefore, the aim of the scoping review is to provide a comprehensive overview of the existing literature of studies using PFMT mobile apps. This review seeks to address the following objectives: (1) identify the BCTs used in PFMT mobile apps, (2) identify the core behaviors of the COM-B targeted by the BCTs in PFMT mobile apps, and (3) compare and explore the levels of PFMT adherence among women using PFMT apps and those receiving usual care.

Through these aims and objectives, this scoping review seeks to contribute to the understanding of the existing interventions within PFMT apps to illuminate their potential role in supporting and promoting adherence to PFMT among women.

## Methods

### Rationale

The scoping review was conducted in accordance with the PRISMA-ScR (Preferred Reporting Items for Systematic Reviews and Meta-Analyses Extension for Scoping Reviews) [29,30]. To develop clearly defined research questions, the process created by the Joanna Briggs Institute was followed [31].

### Search Strategy and Inclusion

The following electronic bibliographic databases were searched: MEDLINE, CINAHL, Scopus, Web of Science, and PEDro, along with CENTRAL. Studies were also identified from reference searching of systematic reviews. The search dates ranged from 2006 to January 2023. Studies before the year 2006 were excluded because mobile technology was unable to support mobile apps and the concept of self-management had not been widely used before this date [32].

Search terms followed the “patient or population, intervention, comparison, and outcomes” model and included the following terms: “women,” “mobile apps,” “mHealth,” “pelvic floor muscle,” “kegel,” “compliance,” and “adherence.” Multimedia Appendix 1 contains a list of all the search terms. A title and abstract search was conducted in January 2023. The full search strategy is presented in Multimedia Appendix 2. The a priori protocol was developed and published on PROSPERO (International Prospective Register of Systematic Reviews) [33]. The “patient or population, intervention, comparison, and outcomes” eligibility criteria for this study are presented in Textbox 1.

The exclusion criteria were as follows: nonexperimental quantitative studies, qualitative studies, studies involving men, case studies, web-based interventions, and adolescent women aged 18 years or younger. Web-based interventions were excluded because of the technological advances in phones to keep up with user activity. Only studies written in English were included. Unpublished studies were not sought. For the review, adherence outcomes were self-reported exercise adherence, in-app adherence measures, and self-efficacy. There was no restriction on what measurement tools could be used to measure adherence. Studies were excluded if they did not measure these concepts.

**The “patient or population, intervention, comparison, and outcomes” eligibility criteria.**

**Eligibility criteria**

- Population: women aged 18 years or older, either asymptomatic or symptomatic of stress urinary incontinence, urge urinary incontinence, or mixed urinary incontinence.
- Intervention: mobile apps for urinary incontinence that support pelvic floor muscle training.
- Comparison: standard care, supervised physiotherapy or home-based training, education delivered through apps, waitlist control, and no treatment.
- Outcome: primary outcome (adherence to pelvic floor muscle training) and secondary outcome (self-efficacy).

### Data Extraction

**Figure 1:**
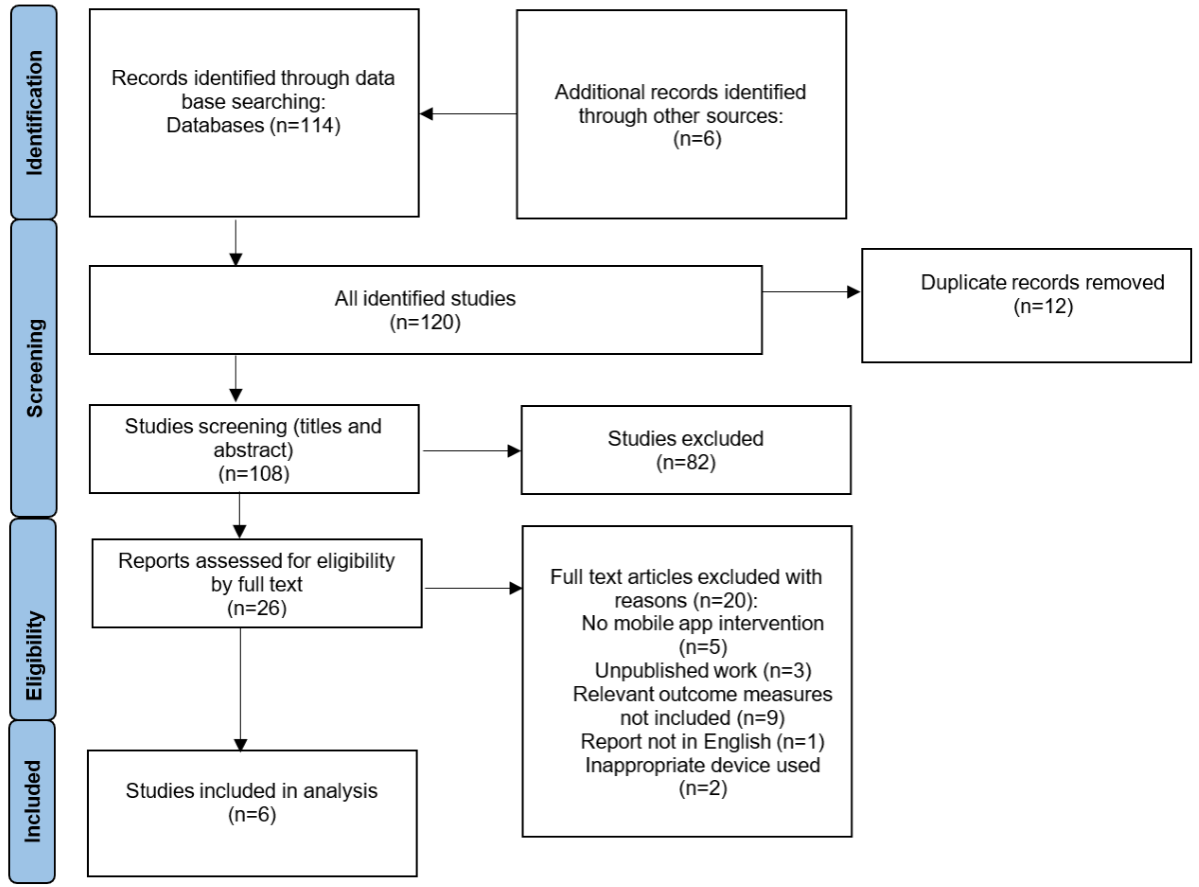
PRISMA-ScR (Preferred Reporting Items for Systematic Reviews and Meta-Analyses Extension for Scoping Reviews) flowchart of study selection.

The integrity of the review was demonstrated using the PRISMA-ScR (Figure 1). Studies were retrieved using a search strategy, and those from additional sources were screened based on their titles and abstracts by the first author (RCH) to determine if they met the inclusion criteria mentioned above. Studies that fulfilled the inclusion criteria were retrieved and independently assessed by the authors (RCH and CJC). The researchers were not blinded to each other’s decisions.

A standardized data extraction form was used by 2 authors to independently extract data on the name and location of the study, study participants, study design, PFMT program, description of PFMT mobile app features, outcome measures, and results in the eligible studies.

Discrepancies between authors RCH and CJC were identified and resolved through discussion. The data were recorded in an Excel (Microsoft Corp) spreadsheet (Multimedia Appendix 3 [34-39]).

### Data Synthesis

Adherence data were analyzed descriptively. A meta-analysis was not possible because of the clinical and methodological diversity in the included studies [40]. The aim of the analysis was to explore differences in adherence levels between the intervention and comparator groups.

BCTs identified in PFMT mobile apps were mapped using the COM-B model [22]. To determine which BCTs used in the intervention group target which core behavior of the COM-B model, the step-by-step mapping guidance to design behavioral interventions using the COM-BCW was used in reverse [22,24]. Rather than designing an intervention, the reverse allowed the identification of each core behavior from each BCT using the theoretical domains framework linked with the COM-B [22]. The 5-step process went as follows:

1. Descriptions of the intervention group were extracted.
2. BCTs were identified based on the above descriptions using BCT Taxonomy Version 1 [22].
3. BCTs were linked to their associated theoretical domains according to the theoretical domains framework [22].
4. Each of the theoretical domains was then linked to the COM-B model.
5. The core element of “capability, opportunity, and motivation” was then identified.

The characteristics of the intervention group were extracted into an Excel spreadsheet (Multimedia Appendix 3 [34-39]).

The description of the PFMT mobile app features and their purpose in the included papers allowed the authors (RCH and CJC) to identify which BCTs they used. The individual PFMT mobile apps were not downloaded. It was assumed that the authors of the included studies followed the correct way to report complex digital interventions and that their description was accurate. The descriptions of the digital interventions were included in the data extraction table (Table 1). All recognized BCTs were included. The BCT identification was completed by authors RCH and CJC. Any discrepancies between the identified BCTs were resolved through discussion. All PFMT mobile apps include one or more BCTs. No BCTs in the taxonomy were excluded based on perceived appropriateness in health and care settings, for example, future punishment.

Using the description of the PFMT mobile app features and their purpose in the included papers, the associated BCT was identified; for example, “daily reminder notification” is associated with “prompts and cues.” Descriptions of PFMT mobile app features with no associated BCT were excluded. The identified BCTs were then grouped and mapped in a Word (Microsoft Corp) table (Table 2).

**Table 1:** Data extraction table of included studies.

Paper	Country	Study participants	Study design	PFMT^a^ program	Description of PFMT mobile app features	Outcome measures
Jaffar et al [38]	Malaysia	26 pregnant women aged between 21 and 39 years	Pilot RCT^b^; app: n=16; control: n=10	A total of 11 contractions a day (10 fast and 1 slow). Length of a sustained contraction is 2-10 seconds. Repeated 3 times a day for 8 weeks.	Educational video; training timer; symptom calendar; daily reminder notification; progress chart; frequently asked questions	Exercise Adherence Rating Scale; Broome Self-efficacy Scale for Practicing Pelvic Floor Exercises Questionnaire
Araujo et al [35]	Brazil	33 women aged between 36 and 67 years	RCT; app: n=17; control: n=16	A total of 4 contractions a day (3 fast and 1 slow). Length of a sustained contraction is 8 seconds. Repeated 2 times a day for 12 weeks.	Visual feedback; reminders; audio feedback with PFMT; self-reported perception of improvement; exercise log	In-app adherence measures; self-reported adherence measure
Kinouchi and Ohashi [39]	Japan	58 postnatal women aged between 31 and 37 years	HCT^c^; app: n=29; control: n=29	A total of 6 contractions a day (fast unspecified and 6 slow). Length of a sustained contraction is 3-6 seconds. Repeated 3 times a day for 12 weeks.	Reminder system	Exercise implementation rate; median training intensity; exercise frequency
Wang et al [36]	China	108 pregnant women aged between 23 and 34 years	RCT; app: n=54; control: n=54	Participants were given the option of either (1) an unspecified number of slow contractions holding for >2 seconds twice a day for 12 weeks, or (2) a total of 150 pelvic floor contractions per day for 12 weeks.	Systematic audio guidance PFMT program; audio reminders; a dynamic graph to support PFMT	Broome Self-efficacy Scale for Practicing Pelvic Floor Exercises Questionnaire
Asklund et al [34]	Sweden	123 women aged between 27 and 72 years	RCT; app: n=62; control: n=61	12 “levels” of PFMT programs (basic and advanced). Repeated 3 times a day for 12 weeks.	Information (on the pelvic floor, SUI^d^, and lifestyle factors); statistics function that calculates the number of exercises performed; reminders; graphic to support PFMT	Self-reported adherence measure
Wadensten et al [37]	Sweden	123 women aged between 31 and 77 years	RCT; app: n=60; control: n=63	12 “levels” of PFMT programs (basic and advanced). Repeated 3 times a day for 12 weeks.	Information (on PFMT, the bladder, psychological topics, and lifestyle advice); reinforcement messages and daily reminders; exercise log; bladder diary	Perceived ability to correctly perform PFMT

^a^PFMT: pelvic floor muscle training.

^b^RCT: randomized controlled trial.

^c^HCT: historical controlled trial.

^d^SUI: stress urinary incontinence.

**Table 2:** The features of pelvic floor muscle training (PFMT) mobile apps and their associated behavior change techniques (BCTs).

Study authors	Intervention features	BCTs used in the intervention group			
		Self-monitoring of behavior	Self-monitoring of outcomes of behavior	Instruction on how to perform behavior	Prompts and cues
Jaffar et al [38]	Educational video, training timer, symptom calendar, daily reminder notification, progress chart, and frequently asked questions		✓	✓	✓
Asklund et al [34]	Information (on the pelvic floor, SUI^a^, and lifestyle factors), statistics function that calculates the number of exercises performed, reminders, and graphic to support PFMT	✓		✓	✓
Kinouchi and Ohashi [39]	Reminder system				✓
Wang et al [36]	Systematic audio guidance PFMT program, audio reminders, and a dynamic graph to support PFMT			✓	✓
Araujo et al [35]	Visual feedback, reminders, audio feedback with PFMT, self-reported perception of improvement, and exercise log	✓	✓	✓	✓
Wadensten et al [37]	Information (on PFMT, the bladder, psychological topics, and lifestyle advice), reinforcement messages and daily reminders, exercise log, and bladder diary	✓	✓	✓	✓

^a^SUI: stress urinary incontinence.

The BCTs in the PFMT mobile apps and usual care groups were then directly linked to their associated theoretical domains [22]. For example, “prompts and cues” are associated with the “environmental context and resources.” The final step involved linking the theoretical domains with the COM-B components; for example, “environmental context and resources” is linked with “physical opportunity.” This process allowed the core behavior (the BCT was targeting) to be identified according to the process described in the BCW, a guide to designing interventions [22], which is displayed in Table 3.

Identified BCTs were narratively synthesized following the guidance for conducting systematic scoping reviews [41].

**Table 3:** The identified behavior change techniques (BCTs) and how they can be linked to a core behavior of the Capability, Opportunity, Motivation, Behavior (COM-B) using the theoretical domains framework (TDF).

Behavior change techniques used in different studies	Associated TDF domain	COM-B component identified in behavior analysis	COM core behavior
Prompts and cues [34-39]	Environmental context and resources	Physical opportunity	Opportunity
Self-monitoring of behavior [34,35,37]	Knowledge	Psychological capability	Capability
Self-monitoring of outcomes of behavior [35,37,38]	Knowledge	Psychological capability	Capability
Instruction on performing behavior [34-38]	Knowledge	Psychological capability	Capability

## Results

### Study Inclusion

Figure 1 details the process of selecting studies according to PRISMA-ScR standards. A total of 114 studies were retrieved and screened against the inclusion and exclusion criteria. A total of 6 studies met the inclusion criteria, including 4 randomized controlled trials (RCTs) [34-37], 1 pilot RCT [38], and 1 historical controlled trial [39].

### Characteristics of Selected Studies

The studies included were published between 2017 and 2022. A total of 2 studies were published in Sweden [34,37], and the rest were published in Brazil [35], China [36], Japan [39], and Malaysia [38]. Sample sizes ranged from 26 to 129, for a total of 471 participants. All studies were treatment studies [34-39], and no prevention studies were found. A detailed summary of all the characteristics of the studies selected can be found in Table 1.

### Sample Population

Women aged between 21 and 72 years were recruited in the included studies, with half of the studies including women aged 40 years or younger [36,38,39]. Women included in all 6 studies had access to a smartphone [37-39], and 3 studies required internet access [34,37,38]. A total of 2 studies involved pregnant women between 26 and 32 weeks of gestation [36,38], and 2 studies recruited women following recent vaginal delivery [36,39]. A total of 4 studies recruited women who had symptoms of SUI [34-36,38]. A total of 3 studies recruited women with mixed urinary incontinence [35,37,38], and 1 study recruited women with urge urinary incontinence [38].

### Intervention and Control

The study length ranged from 2 to 6 months. In 4 studies [34,37-39] PFMT was recommended 3 times per day, and in 1 study, PFMT was recommended twice a day [35]. Alternatively, in 1 study [36], participants were given the option of completing PFMT twice a day or completing 150 pelvic floor muscle contractions a day [36]. The number of fast contractions varied between 0 and 10, the number of slow contractions between 1 and 6, or training time was recommended up to 15 minutes [36]. A total of 2 studies [34,37], stated their intervention group had different “combinations” of PFMT programs participants could use, but this was not expanded. The variety of PFMT training programs and modes of delivery used in the included studies highlights the lack of standardization of PFMT programs and could potentially influence the outcomes. In addition, a total of 3 studies [36,38,39] used a higher PFMT frequency and duration than recommended [9].

A total of 2 studies [35,39] used health care professionals to provide PFMT information to both the intervention and control groups. A specialist physiotherapist taught PFMT using surface electromyography and pelvic floor examination in 1 study [35]. Midwives taught participants through verbal instruction and written information in the other [39]. In comparison, 1 study used trained members of the research team to deliver PFMT [36]. In this study, all participants were given 2 sessions of a 45-minute presentation on pelvic floor education and one-on-one PFMT practice at the start and just before the end of the study [36]. The first presentation focused on the anatomy of the pelvic floor and the PFMT technique. The second focused on the importance of sustaining PFMT [36].

A total of 1 study offered 3 follow-ups with a continuous health care professional that included a pelvic floor examination for all participants [35]. One study offered follow-up telephone contact from a member of the research team once a month to encourage all participants to complete PFMT [36]. In 1 study [38], participants in the controlled group received usual antenatal care from a midwife.

A total of 2 studies provided written PFMT information to the control group [34,35], 1 study [38] postponed the provision of the intervention app until the study was complete, and 2 studies [36,39] provided no additional information. In 1 study [37], the control group received a restricted version of the PFMT mobile app used in the intervention group. A total of 4 studies [34,36,37,39] provided digital support to the intervention group using a PFMT mobile app. In summary, usual care varied between the studies.

It is evident that the variation in PFMT programs, modes of delivery, and levels of professional involvement underscores the lack of uniformity in PFMT across the studies. This makes it difficult to compare the results between studies.

### Outcome Measures

Adherence was the primary outcome measure for 4 studies [35,36,38,39], with the remaining studies reporting it as a secondary outcome [34,37]. The primary outcomes of these studies were the International Consultation on Incontinence Questionnaire for Urinary Incontinence Short Form [42] and the Lower Urinary Tract Symptoms Short Form [42], used in 1 study [34].

The outcome measures used to measure PFMT adherence included the Exercise Adherence Rating Scale [38,43], the Broome Self-efficacy Scale for Practicing Pelvic Floor Exercises Questionnaire [36,44], self-reported adherence using questionnaires [39], or the Visual Analog Scale scores [35]. In one study [35], in-app adherence measures were also used that recorded the number of PFMT protocol repetitions the participants in the intervention groups completed. The findings suggest there was no agreement on how to measure adherence.

### The BCTs Used in PFMT Mobile Apps

The BCTs used in the intervention groups were “self-monitoring of behavior,” “self-monitoring of outcomes of behavior,” “instruction on how to perform behavior,” and “prompts and cues” (Table 2). The findings suggest that a combination of BCTs were used in the intervention group.

The BCT “prompts and cues” were used in all intervention groups [34-39]. In 5 of 6 intervention groups, the BCT “instruction on how to perform behavior” was used [34-38]. A total of 2 intervention groups used all the BCTs mentioned above [35,37], whereas 1 used only “prompts and cues” [39]. In summary, “prompts and cues” were the most frequently used BCT in the intervention groups.

### Core Behaviors of the COM-B Framework Targeted by the BCTs Used in PFMT Mobile Apps

The COM-B core behaviors targeted by the BCTs used in the studies were opportunity and capability (Table 3). Opportunity was targeted using “prompts and cues” in all the intervention groups [34-39]. Capability was the core behavior targeted using the BCTs “self-monitoring of behavior,” “self-monitoring of outcomes of behavior,” and “instruction on how to perform behavior” in the intervention group [34-38]. The findings suggest that the core behavior opportunity was most frequently targeted using “prompts and cues” in the intervention groups.

### PFMT Adherence

The levels of adherence to PFMT were greater in the intervention group compared with the control group, according to 2 studies [34,39]. In 1 study [34], daily PFMT adherence in the intervention group was 40% compared with 3% in the control group, with 60% of the control group reporting “sporadic” PFMT. Conversely, in 1 study [39], daily PFMT adherence was reported at 15 repetitions a day over 7 days in the intervention group compared with once a day over 3 days in the control group. In summary, higher levels of daily adherence to PFMT were observed in the intervention group.

In the control group, PFMT adherence decreased steadily over time [35,36]. In comparison, PFMT adherence increased over time in the control group of another study [38]. Participants in the control group, where adherence increased over time, received a PFMT mobile app at the end of the study period, which may have influenced the results.

## Discussion

### Principal Results

To achieve objective 1, we conducted an analysis of the BCTs used within studies with PFMT mobile app features. Our analysis identified a combination of BCTs with a range of strategies to facilitate adherence to PFMT, including “self-monitoring of behavior,” “self-monitoring of outcomes of behavior,” “instruction on how to perform behavior,” and “prompts and cues.” “Prompts and cues” were the most widely used BCT. To achieve objective 2, we mapped the BCTs used in the studies onto the core behaviors of the COM-B model. This highlighted the central emphasis on the core behavior of opportunity, evident through the prevalence of “prompts and cues” across all intervention groups. Additionally, “self-monitoring of outcomes of behavior” and “instruction on how to perform behavior” were used to address the core behavior capability. The widespread use of “prompts and cues” highlights their role in fostering PFMT engagement by making the most of opportune moments. Furthermore, the incorporation of “self-monitoring of behavior” and “instruction on performing behavior” empowers participants with the necessary knowledge and confidence for effectively executing PFMT. To address objective 3, data on PFMT adherence were extracted from the included studies. This included adherence data from self-efficacy measures and patients’ self-reported adherence measures. The findings suggest higher levels of PFMT adherence were observed in women using PFMT mobile apps; however, there was heterogeneity in the outcome measures used in the included studies, and meta-analysis was not possible.

Digital “prompts and cues” were used by all PFMT mobile apps. Digital “prompts and cues” are a BCT that target the core behavior opportunity according to the COM-B model. Digital reminders are a “persuasive” behavioral intervention that may be valuable when it comes to changing women’s behavior around PFMT, since women commonly associate poor adherence with forgetting to complete PFMT [14,28]. Persuasive behavioral interventions can help women overcome known barriers to PFMT by creating more positive attitudes toward the exercises [28]. These findings are supported by a systematic review that found daily PFMT reminders “stimulate” daily adherence to PFMT in women [21]. Similarly, a systematic review that aimed to determine the persuasive features used in PFMT mobile apps recognized that reminder features were a way of assisting users to remember to perform PFMT, which the authors mapped under the core behavior motivation rather than opportunity [28], which demonstrates how elements of BCT are disputed. In the wider literature, digital reminders in populations with long-term conditions, including diabetes, suggest that digital reminders increase adherence to medication and physical activity [45-47], although none of these papers discuss how behaviors might be influenced. In this review, the results suggest digital PFMT prompts increased daily PFMT adherence in women, and it might be argued that leveraging either or both of the core behaviors of opportunity or motivation. However, further rigorous research is required to explore the effects of digital PFMT prompts on women’s PFMT adherence while controlling other behavioral variables.

Some studies have observed higher levels of PFMT adherence among women using PFMT mobile apps [34,39]. Although the intervention groups reported higher levels of daily adherence, women did not meet the daily PFMT recommendations of 3 times a day [2]. In 1 study [35], women completed PFMT 1.6 times a day on average, and in another [37], only 10% of women completed PFMT 3 times a day. One reason for the lower levels of PFMT adherence may have been the heterogeneity of the outcome measures of adherence that were inconsistently monitored and poorly described [12]. Alternatively, the heterogeneity in PFMT program prescription may have influenced adherence to PFMT, with 3 studies prescribing a higher dosage of PFMT [36,38,39] than recommended [9]. The high frequency and intensity of PFMT recommendations are at odds with the “easy, accessible, social, timely” principles of behavior change [48]. If the exercises and the intensity of the regime appear difficult, it is likely that women are less likely to engage in and adhere to PFMT. This is particularly important to consider given the fact that no prevention studies were identified in the scoping review, and women may find adhering to PFMT even harder if they are asymptomatic.

Women’s adherence to PFMT appeared to decrease around 3 months [35,36,38]. These findings are supported by other studies that have explored adherence rates in people with chronic disease and national guidelines [2,48], suggesting digital prompts may only be valuable in the short term. It is recognized that short-term PFMT adherence is greater than long-term adherence [7]. Previous research indicated that behavior change can take up to a year [49]. In the included studies of this review, there were none that followed up a year postintervention, and therefore we are unable to make a comment on the long-term nature of behavioral change. The decrease in PFMT adherence after 3 months may reflect a normal drop in adherence levels [49] because women notice an improvement in their symptoms and no longer consider the exercises to be beneficial. It has been suggested that “social opportunity” rather than “physical opportunity” (referenced in the COM-B model) is important when it comes to behavioral maintenance, since actions are more likely to be sustained when in line with group norms [49]. Further behavioral research on PFMT adherence in women should increase the length of study to 1 year and explore the role of “social opportunity” on adherence.

Conversely, in the control group of 1 study, adherence increased at 2 months [38]. A potential reason for this is that the control group received “usual antenatal care” that involved contact with health care professionals. The influence of health care experts on exercise adherence is “pivotal,” according to other studies [50]. Arguably, contact and follow-up with a health care professional as a “credible source” may be a valuable BCT in relation to PFMT adherence in women because it holds women accountable to complete PFMT [22]. The use of health care professionals to teach PFMT and follow-up participants in some of the included studies may have led to confounding behavioral factors that influenced individuals’ adherence to PFMT. Future research should consider the influence health care professionals have on participants PFMT adherence when conducting future research on PFMT mobile apps.

### Strengths and Limitations

Using a scoping review methodology, we have been able to identify a breadth of experimental studies investigating the effect of PFMT apps on adherence to PFMT in women as an intervention. To the authors’ knowledge, this study was the first scoping review to identify and discuss specific BCTs embedded within existing features of PFMT mobile apps and to map the core behaviors targeted using the COM-BCW framework. By doing so, this research contributes to a deeper comprehension of the factors influencing PFMT adherence among women and highlights the potential use of PFMT mobile apps, particularly those with digital prompts, to increase adherence to PFMT. Additionally, the study uses the COM-B model to provide a holistic understanding of how capability, opportunity, and motivation are used in the context of PFMT adherence, bridging the gap between theoretical concepts and application that can inform the development and enhancement of interventions targeting PFMT adherence.

A limitation of the study was the way BCTs were assigned to the features of PFMT mobile apps based on descriptions of the apps and app features in the included papers. One BCT was assigned per app feature, and features that did not specifically relate to any of the 93 BCTs were not included. Inaccurate descriptions of mobile app features by the authors of the included studies may have resulted from misattribution or underestimation of the BCTs and therefore misrepresentation of the core behaviors targeted by PFMT mobile apps. The misrepresentation of core behaviors may have made the core behaviors that encourage women to adhere to PFMT unclear. Additional BCTs may have been identified if the PFMT mobile apps were downloaded or original papers around the app development were sought and appraised.

Another limitation of the study was the exclusion of studies without a control group. The exclusion of additional studies, such as observational studies, meant that studies with potentially fuller descriptions of PFMT mobile apps and their intended behavioral mechanisms were not included in the analysis. Arguably, this limits the depth of the analysis in the study by limiting the comparison and corroboration between a greater number of PFMT mobile apps that would have better addressed the first 2 objectives of this study. Alternatively, the inclusion of studies with a control group increased our confidence in the findings that suggest levels of adherence were higher in women using a PFMT mobile app compared to an alternative variable and addressed objective 3.

This review identified the use of mobile apps for the treatment of PFMT and the behaviors being leveraged; however, due to the heterogeneity of the PFMT regimes across the studies, it was not possible to compare. In the future, it will be important to identify the minimum effective training dosage women engage with and why.

### Conclusion

The findings suggest there was a trend in higher levels of PFMT adherence in women using PFMT mobile apps over 3 months. However, there was no consistency across the studies in the PFMT regimes and outcome measures. Digital “prompts and cues” were used to encourage adherence and may be the BCT that leveraged improved adherence. Opportunity was the most targeted core behavior in the intervention groups of the included studies. Further research is required to practically assess whether a future RCT that investigates the effectiveness of digital “prompts and cues” on PFMT adherence in women can be conducted.

## Appendix

Multimedia Appendix 1 Search terms.

Multimedia Appendix 2 Search strategy.

Multimedia Appendix 3 Raw data extraction table of the included studies.

Multimedia Appendix 4 PRISMA-ScR (Preferred Reporting Items for Systematic reviews and Meta-Analyses extension for Scoping Reviews) checklist.

## Abbreviations

BCT: behavior change technique
BCW: behavior change wheel
COM-B: Capability Opportunity Motivation Behavior
COM-BCW: Capability Opportunity Motivation Behavior change wheel
PFD: pelvic floor dysfunction
PFMT: pelvic floor muscle training
PRISMA-ScR: Preferred Reporting Items for Systematic Reviews and Meta-Analyses Extension for Scoping Reviews
PROSPERO: International Prospective Register of Systematic Reviews
RCT: randomized controlled trial
SUI: stress urinary incontinence
VAS: Visual Analog Scale
