# Supplementary material for: Exploring Adherence to Pelvic Floor Muscle Training in Women Using Mobile Apps: Scoping Review
Source: JMIR Mhealth Uhealth. 2023 Nov 30;11:e45947. doi: 10.2196/45947 (PMC10722367; doi:10.2196/45947)
Supplement: Multimedia Appendix 1 [file mhealth_v11i1e45947_app1.docx]

# Appendix 1: Search terms

| Search term | Alternatives |
| --- | --- |
| Women | wom*n or femal* or urinary incontinen* or urge urinary incontinen* or mixed urinary incontinen* or stress urinary incontinen* or overactive bladder |
| Apps | m-health or mobile app or mobile application* or smartphone app* or digital health technolog* or telecare or telehealth or telemedicine |
| Pelvic floor muscle training | pelvic floor muscle training or pelvic floor muscle exercise* or kegel exercise* or kegel* |
| Adherence | adhere* or compliance or sustain* or maintain* or engag* or attend* |
