# Supplementary material for: Exploring Adherence to Pelvic Floor Muscle Training in Women Using Mobile Apps: Scoping Review
Source: JMIR Mhealth Uhealth. 2023 Nov 30;11:e45947. doi: 10.2196/45947 (PMC10722367; doi:10.2196/45947)
Supplement: Multimedia Appendix 2 [file mhealth_v11i1e45947_app2.docx]

# Appendix 2: Search strategy

| S1 | TI wom*n OR female* OR urinary incontinen* OR urge urinary incontinen* OR mixed urinary incontinen* OR stress urinary incontinen* OR overactive bladder |
| --- | --- |
| S2 | AB wom*n OR female* OR urinary incontinen* OR urge urinary incontinen* OR mixed urinary incontinen* OR stress urinary incontinen* OR overactive bladder |
| S3 | TI mhealth OR mobile health OR m-health OR mobile app OR mobile application* OR smartphone app* OR digital health technolog* OR telecare OR telehealth OR telemedicine |
| S4 | AB mhealth OR mobile health OR m-health OR mobile app OR mobile application* OR smartphone app* OR digital health technolog* OR telecare OR telehealth OR telemedicine |
| S5 | TI pelvic floor muscle training OR pelvic floor muscle exercise* OR kegel exercise* OR kegel |
| S6 | AB pelvic floor muscle training OR pelvic floor muscle exercise* OR kegel exercise* OR kegel |
| S7 | TI adhere* OR compliance OR sustain* OR maintain* OR engag* OR attend* |
| S8 | AB adhere* OR compliance OR sustain* OR maintain* OR engag* OR attend* |
| S9 | S1 OR S2 |
| S10 | S3 OR S4 |
| S11 | S5 OR S6 |
| S12 | S7 OR S8 |
| S13 | S9 AND S10 AND S11 AND S12 |
